# What Do Israeli Osteoporotic Men Know and Do about Their Disease?

**DOI:** 10.4061/2011/719862

**Published:** 2011-06-14

**Authors:** Offer Emanuel Edelstein

**Affiliations:** School of Social Work, University of Haifa, Haifa 31905, Israel

## Abstract

*Aims*. The aims of the current study were to evaluate the level of knowledge about osteoporosis among osteoporotic men and to assess the correlations with their health behaviors. *Method*. A convenience sample of 100 osteoporotic men (mean age 63) attending the bone and mineral clinic at a major medical center in Israel was recruited in 2004. Participants were interviewed by phone using an adapted version of the Facts on Osteoporosis Quiz (FOOQ). Participants were also asked to report on their daily calcium intake and participation in physical activities. *Results*. The overall level of knowledge about the disease demonstrated by the participants was moderate. Higher education, older age, and fewer fractures were correlated with a higher level of knowledge. In addition, higher levels of education and knowledge were correlated with higher calcium intake. Lastly, a higher knowledge level, older age, and fewer fractures were correlated with higher participation in physical activities. *Conclusions*. Given the correlations between health behaviors and the level of knowledge among osteoporotic men, intervention programs should be used to evaluate and improve knowledge about osteoporosis, especially among less educated patients.

## 1. Introduction

Osteoporosis is a skeletal disorder characterized by compromised bone strength predisposing a person to an increased risk of fracture. Bone strength reflects the integration of two main features: bone density and bone quality [1]. While osteoporosis is frequently regarded as a “women's disease,” up to two million men in the US alone suffer from the disease [2]. A recent study showed that one in three osteoporotic fractures after the age of 50 occurs in men [3]. More importantly, men have a higher mortality rate from hip fractures and a lower frequency of screening and treatment as compared to women [4, 5]. As such, osteoporosis should be treated as a disease that affects both genders. 

Although osteoporosis is an incurable disease, there are several steps that can be taken in order to control bone loss, including adequate calcium and vitamin D intake, regular physical exercise, and pharmacological treatment [6, 7]. Research in osteoporosis has paid considerable attention to the level of knowledge among women and its associations with health behaviors. For example, Werner and colleagues [8] found that women demonstrated a higher level of knowledge regarding the role of physical activity and calcium intake in the prevention of osteoporosis, but knew less about the role of genetics, caffeine intake, alcohol consumption, and smoking as risk factors. In addition, a higher level of knowledge was found to be associated with such health behaviors as greater calcium intake and regular performance of physical activities [8–12]. 

Despite the fact that osteoporosis is a major health concern among men, little research attention has been paid to evaluating the associations between knowledge and health behaviors in this population. The limited number of studies among men shows that they demonstrate a low level of knowledge about the disease [13–15]. To the best of the author's knowledge, only one study [16] has evaluated the correlations between the level of knowledge about osteoporosis and health behaviors among men. Although no direct associations were found between these variables, it must be noted that only a small portion of the participants in this study and the other studies mentioned above were diagnosed as osteoporotic [15, 16]. Hence, the ability to generalize from their findings to the population of osteoporotic men is questionable. The aims of the current study were to evaluate the level of knowledge about osteoporosis among osteoporotic men and to assess the correlations with their health behaviors.

## 2. Method

### 2.1. Participants

A convenience sample of 100 osteoporotic men attending the bone and mineral clinic at a major medical center in Israel was recruited in 2004. The sociodemographic and health characteristics of the sample are presented in Table 1. The participants' mean age was about 63, the vast majority was married, and their level of education and income were relatively high. Only one-third of the participants had an osteoporotic fracture. The mean *t*-score was −2.4 (S.D. = 1.5) at the lumbar spine and −2.7 (S.D. = 0.8) at the femoral neck.

### 2.2. Measures

The following measures were used to collect data for the present study. 

#### 2.2.1. Health Behaviors

Two types of health behaviors were assessed, namely, calcium intake and participation in physical activities.

**(a) Calcium Intake**

Similar to other studies [8, 17], the participants' calcium intake was measured by a self-report questionnaire on the number of glasses of milk, servings of yogurt, slices of hard cheese, and cups of white cheese consumed daily, as well as the type and amount of soy products and calcium supplements consumed. Scores were calculated for calcium intake in milligrams, based on the information presented in the NIH Web site [18] and adapted for Israel. The content validity of the calcium intake measures was provided by two Israeli experts in the field of osteoporosis.

**(b) Physical Activity**

Participation in physical activity was assessed by asking whether participants regularly engaged in physical activities, which type of activities they engaged in (e.g., walking, swimming, weight-bearing exercises, and home exercises), the number of times a week, and for how many minutes each time. A total score of the number of minutes engaged in all physical activities was calculated.

#### 2.2.2. Knowledge Regarding Osteoporosis Disease

Participants' knowledge about osteoporosis was assessed by an adapted version of the Facts on Osteoporosis Quiz (FOOQ) [19]. The original quiz consists of 24 true or false statements related to risk factors and preventive behaviors associated with osteoporosis. For this study, one item referring to African-American women was deleted and four statements regarding the incidence of fractures and risk factors related to men were added. Similar to other studies [8, 9], a “Don't know” response was added in order to reduce the likelihood of guessing. Each item was coded as 0 for incorrect and “Don't know” responses or 1 for the correct answer. Internal consistency for this study was Cronbach *α* = 0.77.

#### 2.2.3. Demographic and Health Data

This information included gender, age, education level, income, and marital status. In addition, data were collected from the participants' medical records regarding the number of previous fractures, time since the last fracture, and bone density scan scores.

### 2.3. Procedure

A computerized search of medical records was performed by the physician in order to locate men who had been treated for osteoporosis during the year preceding the study. A total of 115 patients were approached by the physician and presented with the aims of the study. A response rate of 87% was obtained, resulting in a final sample size of 100 participants. Once the participants signed informed consent forms, they were contacted by the researcher and were interviewed on the phone using a structured questionnaire. 

#### 2.3.1. Ethical Considerations

The research protocol was approved by the Helsinki committee of the medical center. All participants signed informed consent forms. In order to protect the participants' privacy, the data obtained from their medical records and interviews were coded anonymously to a password-protected file.

#### 2.3.2. Statistical Analysis

The statistical analysis included descriptive statistics (means, standard deviations, percentages) to describe the sample and the main study variables (knowledge level, health behaviors, and background data). Pearson's correlations were calculated to assess the correlations between health behaviors, level of knowledge, and background data.

## 3. Results

### 3.1. Level of Knowledge about Osteoporosis

Table 2 presents the percentages of participants who correctly responded to the knowledge items, followed by the mean, median, and range scores for the overall index of knowledge. The overall level of knowledge demonstrated by the participants was moderate. According to the median score, half of the men responded correctly to 66.6% of the statements in the overall index. The highest level of knowledge was found for the items related to the role of physical activities and calcium intake in preventing osteoporosis. However, participants showed lower knowledge for the items related to caffeine intake, alcohol consumption, and smoking as risk factors for osteoporosis. The lowest level of knowledge was found for the items related to the incidence of osteoporotic fractures in men and in regard to hypogonadism as a risk factor.

### 3.2. Health Behaviors

Table 3 presents the means of daily calcium intake and weekly participation in physical activities. As shown in the table, participants reported an average of 842 mg of daily calcium intake and an average of 120 minutes per week for engagement in physical activities. 

Thirty percent of the participants reported that they do not engage in any kind of physical activity. Of the 70% who did report on regular participation in physical activities, 52% reported on walking, 9% reported on swimming, 6% reported on weight-bearing exercises, and 3% reported on daily performance of home exercises.

### 3.3. Correlations between Level of Knowledge, Sociodemographic/Clinical Characteristics, and Health Behaviors

Several statistically significant correlations were found between the participants' sociodemographic/clinical characteristics, their level of knowledge, and their health behaviors. The participants' level of knowledge about osteoporosis was positively correlated with education level and age (*r* = .26, *P* < .01; *r* = .21, *P* < .05, resp.) and was negatively correlated with the number of fractures (*r* = −.19, *P* < .05). Knowledge level and education were positively correlated with calcium intake (*r* = .36, *P* < .0001; *r* = .25, *P* < .05, resp.). Knowledge level and age were positively correlated with participation in physical activities (*r* = .47, *P* < .0001; *r* = .24, *P* < .05, resp.). Finally, the number of fractures was negatively correlated with participation in physical activities (*r* = −.31, *P* < .001).

## 4. Discussion

The current study had two main objectives: to evaluate the level of knowledge about osteoporosis in osteoporotic men and to assess its correlations with health behaviors. Overall, participants demonstrated a moderate level of knowledge about the disease. However, in comparison to other studies among men [13, 15], participants in the present study had a higher level of knowledge. This may be attributable to the fact that while other study samples were recruited from the general population and were largely not osteoporotic, the current sample was recruited from a bone and mineral disorders clinic and participants were diagnosed as osteoporotic. 

Participants showed a high level of knowledge regarding the role of physical activities and calcium intake in preventing osteoporosis. This finding is encouraging, as these activities are major countermeasures against bone loss [6, 7]. However, a lower level of knowledge was found regarding such behaviors as caffeine intake, alcohol consumption, and smoking, which are considered to be risk factors for osteoporosis. Since alcohol consumption and smoking are attributed to “masculine” behaviors [20], the practical meaning of these findings is that health professionals should pay closer attention to mens' knowledge about the effects of these behaviors on bone mass. The lowest level of knowledge was found in regard to the incidence of osteoporotic fractures in men. This finding is rather surprising, as it is reasonable to assume that osteoporotic patients would demonstrate a higher level of knowledge on this matter. The fact that only one-third of the participants had an osteoporotic fracture may serve as an explanation. 

Regarding the second aim of the study, that is, to assess the correlations between the level of knowledge about osteoporosis and the health behaviors of osteoporotic men, the findings support those of studies among women [8–12]. Indeed, a higher level of knowledge was correlated with higher participation in regular physical activities and calcium intake. However, the findings contradict those of Doheny and colleagues [16] showing no direct associations between knowledge level and health behaviors. A possible explanation for this contradiction may be related to the fact that Doheny and colleagues [16] included in their model other variables, such as health beliefs, which were more powerful predictors for these behaviors. Furthermore, other studies showed that improving knowledge alone did not change behavior [21–23]. Since there is very limited data on these correlations among men, future studies should address this discrepancy by incorporating both knowledge and health beliefs assessments in their models. 

Several interesting correlations were found in the present study. For instance, a positive correlation was found between the level of knowledge about osteoporosis and the level of education. As suggested in other studies [8, 9], greater access of highly educated patients to such information sources as professional journals and the Internet may be related to their level of knowledge. 

Higher age was found to be positively correlated with level of knowledge and participation in regular physical activities. The tendency towards later onset of osteoporosis in men [24] may explain why older patients sought out more comprehensive knowledge and concentrated their efforts on engaging in more physical activities. In addition, a lower number of fractures were correlated both with a higher level of knowledge and higher participation in physical activities. The severe impact that osteoporotic fractures have on patients' mobility [25] and the major role that physical activities play in preserving bone mass [7] may account for this finding. 

Finally, the participants' reported mean of 120 minutes of weekly engagement in physical activities is encouraging since it meets the recommended level of physical activity for maintaining bone health [2]. However, only 6% of the participants reported on regular performance of weight-bearing exercises, which are specifically recommended for osteoporotic patients [2]. This finding calls for health professionals to emphasize the contribution that this kind of activity makes to preserving bone mass. Furthermore, the mean amount of calcium reported by the participants was substantially under the recommended 1200 mg for men over the age of 50 [2, 26]. This finding is puzzling given that the participants demonstrated the highest level of knowledge on items related to calcium intake. A possible methodological explanation for this contradiction may be related to the self-report method that was used in the study. Participants were asked to report on their intake of dairy products and calcium supplements during the week preceding the interview. It is possible that they simply underestimated the amount of products consumed and reported on lower calcium intake. As calcium intake is a major step towards protecting against bone mass deterioration, health professionals should emphasize the importance of adequate calcium intake for preserving bone mass. 

Two main limitations regarding the present study should be noted. First, due to the use of a convenience sample, the results of the present study apply to this specific clinical setting and do not allow generalization to all osteoporotic patients in Israel. Second, the cross-sectional nature of the present study calls for caution when interpreting the findings. Despite its limitations, this study makes an important contribution to expanding our knowledge about osteoporosis and the correlations between level of knowledge, performance of regular physical activities, and calcium intake among osteoporotic men. The findings stress the need for health professionals to consider using intervention programs to evaluate and improve the level of knowledge about osteoporosis, especially among less educated patients.

## Figures and Tables

**Table 1:** Sociodemographic and health characteristics (*n* = 100).

Sociodemographic characteristics	
Mean age (S.D.)	62.9 (11.3)
Mean years of education (S.D.)	13.6 (11.3)
Marital status (%)	
Married	85
Income (%)	
400–5999 NIS	31
6000+ NIS	69

Health characteristics	

Had an osteoporotic fracture (%)	33
Mean (S.D.) *t*-scores in lumbar spine	−2.4 (1.5)
Mean (S.D.) *t*-scores in femoral neck	−2.7 (0.8)

**Table 2:** Percentage of participants responding correctly to knowledge items (*n* = 100).

Item	% of participants correctly responded
(1) One in four women over the age of 60 will develop osteoporosis*	65
(2) One of every 3-4 fractures in the femoral neck occurs in men^†∗^	41
(3) Heredity does not play a role in osteoporosis	65
(4) Early menopause, such as hysterectomy, is not a risk factor for osteoporosis	48
(5) High caffeine intake (more than two cups per day) increases the risk of osteoporosis*	35
(6) A lifetime low intake of calcium will increase the risk of osteoporosis*	93
(7) Hypogonadism is not a risk factor for osteoporosis^†^	43
(8) Smoking is not a risk factor for osteoporosis	47
(9) 20–25% of all osteoporotic fractures occur in men^†∗^	40
(10) Weight-bearing exercise such as walking can help prevent osteoporosis*	98
(11) After age 40, it is too late for people to increase their calcium intake to prevent osteoporosis	65
(12) There is no treatment for osteoporosis once you develop it	89
(13) After menopause, osteoporosis may be slowed down by taking estrogen*	36
(14) All individuals lose bone mass after 40 years of age*	62
(15) Men can be totally cured from osteoporosis once they develop it^†^	71
(16) Normally, bone loss slows down after menopause	64
(17) A diet high in calcium throughout life can help prevent osteoporosis*	92
(18) Women over 40 need about 1500 mg of calcium*	67
(19) There is no way to prevent osteoporosis	72
(20) Dairy products are a major source of calcium*	95
(21) It is normal for bone loss to continue throughout life*	85
(22) Active individuals are at higher risk for osteoporosis than inactive individuals	90
(23) Alcohol abuse is not linked to the incidence of osteoporosis	60
(24) A risk factor for osteoporosis is having a mother with it*	78
(25) Young women need the equivalent in calcium of a glass of milk a day to prevent osteoporosis	71
(26) Inactivity increases the risk of osteoporosis*	97
(27) Thin women are more often affected by osteoporosis than heavy ones*	45

*Overall knowledge *	
Mean (S.D.)	18.1 (4.3)
Median	18
Range	6–27

*item is a true statement; ^†^denotes new items, not included in the original FOOQ.

**Table 3:** Mean and standard deviations of calcium intake and participation in physical activities (*n* = 100).

Health behavior	Mean	S.D.	Range
Calcium intake (mg/day)	842.5	387.4	0–1771
Physical activities (min/week)	120.5	111.3	0–600
